# Requirement of β1 integrin for endothelium-dependent vasodilation and collateral formation in hindlimb ischemia

**DOI:** 10.1038/s41598-019-53137-x

**Published:** 2019-11-15

**Authors:** Carina Henning, Anna Branopolski, Dominik Schuler, Dimitrios Dimitroulis, Patrik Huelsemann, Christopher Nicolaus, Roberto Sansone, Jelle Ludolf Postma, Daniel Eberhard, Ferdinand Le Noble, Malte Kelm, Eckhard Lammert, Christian Heiss

**Affiliations:** 1 Institute of Metabolic Physiology, Heinrich Heine University, Duesseldorf, Germany; 2 Division of Cardiology, Pulmonology, and Vascular Medicine, Medical Faculty, University Duesseldorf, Duesseldorf, Germany; 3 Center for Advanced Imaging, Heinrich Heine University Duesseldorf, Duesseldorf, Germany; 4 Institute for Zoology, Karlsruhe Institute of Technology, Karlsruhe, Germany; 5 Institute for Vascular and Islet Cell Biology, German Diabetes Center (DDZ) - Leibniz Center for Diabetes Research, Duesseldorf, Germany; 6 Department of Clinical and Experimental Medicine, Faculty of Health and Medical Sciences, University of Surrey, Guildford, United Kingdom; 7 Surrey and Sussex Healthcare NHS Trust, East Surrey Hospital, Redhill, United Kingdom

**Keywords:** Angiogenesis, Blood flow

## Abstract

An acute increase in blood flow triggers flow-mediated dilation (FMD), which is mainly mediated by endothelial nitric oxide synthase (eNOS). A long-term increase in blood flow chronically enlarges the arterial lumen, a process called arteriogenesis. In several common human diseases, these processes are disrupted for as yet unknown reasons. Here, we asked whether β1 integrin, a mechanosensory protein in endothelial cells, is required for FMD and arteriogenesis in the ischemic hindlimb. Permanent ligation of the femoral artery in C57BL/6 J mice enlarged pre-existing collateral arteries and increased numbers of arterioles in the thigh. In the lower leg, the numbers of capillaries increased. Notably, injection of β1 integrin-blocking antibody or tamoxifen-induced endothelial cell-specific deletion of the gene for β1 integrin (*Itgb1*) inhibited both arteriogenesis and angiogenesis. Using high frequency ultrasound, we demonstrated that β1 integrin-blocking antibody or endothelial cell-specific depletion of β1 integrin attenuated FMD of the femoral artery, and blocking of β1 integrin function did not further decrease FMD in eNOS-deficient mice. Our data suggest that endothelial β1 integrin is required for both acute and chronic widening of the arterial lumen in response to hindlimb ischemia, potentially via functional interaction with eNOS.

## Introduction

Arteries can adapt to changes in blood flow and the vascular endothelium is crucially involved in the fundamental regulation of blood flow to ensure the matching of demand and supply of tissues with oxygen and nutrients. For example, after transient ischemia and during situations of increased oxygen and nutrient demand (e.g., during physical exercise), arterial blood flow increases^1^. As a response to increased shear forces during reactive hyperemia, healthy arteries dilate in the presence of oxygen primarily via release of nitric oxide (NO), a process called flow-mediated vasodilation (FMD)^2^. Long-term increases in blood flow result in a structural enlargement of the arterial lumen. It is known for a long time that endurance training leads to enlargement of femoral arteries in athletes^3^. While the mechanisms are unknown, this structural adaptation involves the outward remodelling along with proliferation of endothelial cells and vascular smooth muscle cells (SMC), monocyte recruitment, and extracellular matrix turnover^4^. The latter processes result in larger vascular lumens and increased numbers of arterioles and capillaries^1^, allowing more blood to flow through the skeletal muscles of the limbs, thus alleviating hypoxia in situations of increased demand for oxygen and nutrients^1^.

In the setting of ischemic diseases, an occlusion of a major feed artery results in downstream dilation of resistance arteries, leading to rerouting of blood flow through pre-existing collateral arteries. In response to the increase in blood flow and shear stress, the collateral arteries dilate (acute response), and, after several days, start to show signs of structural remodelling; this process is referred to as ‘arteriogenesis^4–6^. Two distinct types of adult arteriogenesis exist that result in restoration of blood flow towards the compromised hypo-perfused tissue; that is (i) the enlargement of pre-existing collateral arteries (or *classical* arteriogenesis) and (ii) the process of arterialization of capillaries (or *de novo* arteriogenesis)^4,7^. Remodelling of pre-existing collaterals involves shear stress-dependent activation of endothelial nitric oxide synthase (eNOS), leading to their acute vasodilation^6,8–11^. Vasodilation, in turn, increases circumferential wall stress which then promotes growth and enlargement of the media layer which is supported by recruited monocytes^4,12^. Mechanical stress induces smooth muscle cell proliferation involving a switch in cellular phenotype from a contractile to a proliferative one. The critical role of NO is demonstrated by a markedly lowered arteriogenesis and vessel rarefaction in eNOS (or *Nos3)*-deficient mice^13,14^. In contrast, *de novo* arteriogenesis is due to capillary arterialization^15^. It is a poorly understood process that is believed to be largely driven by endothelial cells^16^, involves change in endothelial cell (EC) fate, and acquisition of a medial layer^4^. Besides arteriogenesis taking place in upstream regions of the ischemic hindlimb, which are less exposed to prolonged hypoxia, more distal regions show increased angiogenesis, a process driven by local skeletal muscle hypoxia activating the HIF-1α-VEGF-A pathway (independent of shear stress)^17^.

How an increased shear stress is sensed is not fully understood, but VE-Cadherin and VEGF receptor-2 as well as Klf2 play critical roles^18,19^. Furthermore, another possible mechanosensory protein on EC could be β1 integrin, a membrane-anchored subunit of many integrins^20,21^. Loss- and gain-of-function studies have shown that endothelial β1 integrin is involved in vascular lumen formation of arteries, angiogenesis, inflammatory processes, and vessel wall remodelling^22–24^. β1 integrin appears to be essential for blood vessel formation during embryonic development as well as for postnatal vascular remodelling, smooth muscle vasomotor control, and wound healing^22–24^. Furthermore, it regulates endothelial cell polarity and arteriolar lumen formation^25^. More recent literature showed that endothelial β1 integrin is required for the formation of stable, non-leaky blood vessels and has an acute function in vessel growth and maturation^26^. While homozygous deletion of *Itgb1* is embryonically lethal, mice with a heterozygous endothelial cell-specific deletion showed abnormal vascular remodelling in response to changed blood flow after external carotid artery ligation^23^. In a rabbit hindlimb ischemia model, it was also demonstrated that α5β1 integrin was upregulated after increases in fluid shear stress in collaterals^27^. A recent *in vitro* study suggests that β1 integrin may not only function in EC via interactions with components of basement membrane, but that it may also trigger shear stress-mediated activation of eNOS at the apical EC surface^28^. Here, we provide evidence that β1 integrin plays an important role in both FMD and arteriogenesis in the thigh during hindlimb ischemia (HI).

## Results

### Blocking β1 integrin function abrogates arteriogenesis and angiogenesis

**Figure 1:**
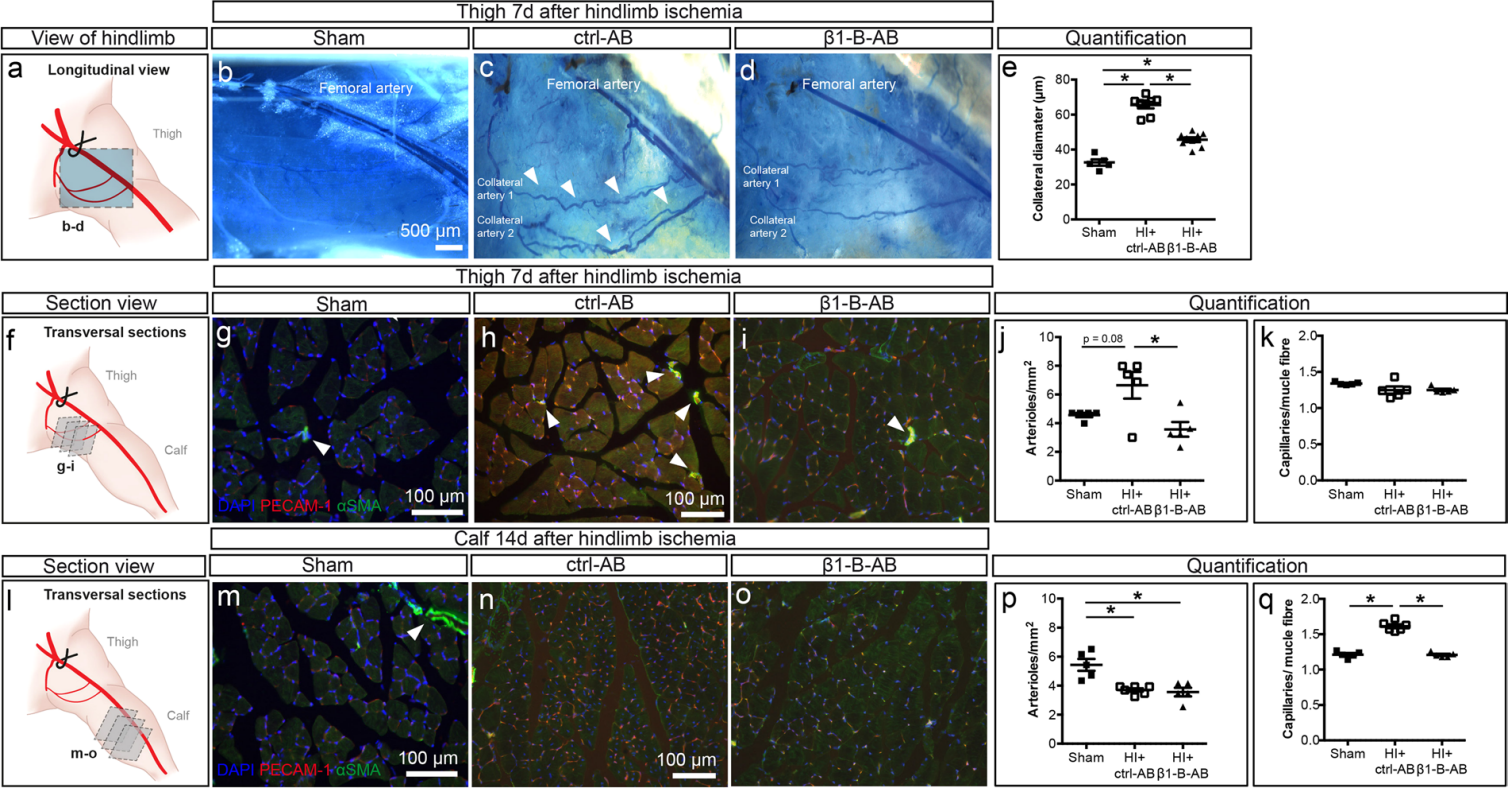
Attenuation of *de novo* arteriogenesis in the thigh and angiogenesis in the calf by β1 integrin blocking antibodies following femoral artery ligation. (**a–e**) Collateral expansion as observed 7 days after femoral artery (FA) ligation. (**a**) Schematic drawing of experimental setup. (**b–d**) Brightfield microscopic image of the thigh, FA was perfused with Pu4 resin (blue): (**b**) thigh of sham operated mice, (**c**) mice after 7 days of hindlimb ischemia (HI) treated with control antibody (HI + ctrl-AB), and (**d**) β1 integrin blocking antibodies (HI + β1-B-AB). (**e**) Quantification of the collateral diameters after 7 days of HI in sham operated mice (n = 5), mice treated with ctrl-AB (n = 8) or β1-B-AB (n = 9). **(f–k)** Analysis of *de novo* arteriogenesis and angiogenesis, in thigh muscles 7 days after HI. **(f)** Schematic drawing of experimental setup. **(g–i)** Fluorescence microscopy images of arterioles and capillaries in thigh muscle stained for cell nuclei (DAPI, blue), endothelial cells (PECAM-1, red) and smooth muscle cells (α-SMA, green) in **(g)** sham operated animals (n = 5), **(h)** mice after 7 days HI with ctrl-AB (n = 5), and **(i)** β1-B-AB (n = 5). **(j** + **k)** Quantification of **(j)** arterioles per mm^2^ and **(k)** capillaries per muscle fibre. **(l–q)** Analysis of *de novo* arteriogenesis and angiogenesis in calf muscles 14 days after HI. **(l)** Schematic drawing of experimental setup. **(m–o)** Fluorescence microscopy images of arterioles and capillaries in calf muscle stained for cell nuclei (DAPI, blue), endothelial cells (PECAM-1, red) and smooth muscle cells (α-SMA, green) in **(m)** sham operated mice (n = 5), **(n)** mice after 14 days HI with ctrl-AB (n = 6), and **(o)** β1-B-AB (n = 5). **(p** + **q)** Quantification of **(p)** arterioles per mm^2^ and **(q)** capillaries per muscle fibre. *All values are mean* ± standard error of the mean (SEM), statistical significance was determined using one-way ANOVA with Tukey post-hoc test, *p ≤ *0.05*.

To study whether β1 integrin plays a role in arteriogenesis under conditions of chronically increased collateral blood flow, we induced HI by total ligation of the femoral artery (FA) in WT mice treated with and without β1 integrin blocking antibodies as well as in gene-deficient mice (Supplementary Fig. S1). While the HI led to significantly decreased perfusion of the thigh and calf, the thigh recovered to baseline perfusion within 3 days, while it remained decreased in the calf (Supplementary Fig. S2). Furthermore, endothelial β1 integrin expression increased in the calf, while only showing a small trend towards higher expression in the thigh (Supplementary Fig. S3), consistent with a previous report showing that hypoxia via HIF-1α can induce β1 integrin expression^29^. Two groups of WT animals received either β1 integrin blocking antibodies or isotype-matched control antibodies (Supplementary Fig. S1a). In the control group, native collateral diameter was increased at day 7 post-ligation as compared to sham-operated/unligated animals (Fig. 1a–e). Integrin antibody treatment significantly inhibited this collateral diameter increase (Fig. 1e), suggesting a functional role for β1 integrin.

Furthermore, FA ligation led to differential microvascular adaptations in the thigh (Fig. 1f–k) compared to the calf (Fig. 1l–q). In the thigh muscles, we observed a higher number of arterioles consistent with *de novo* arteriogenesis (Fig. 1j). In contrast, the number of capillaries was unchanged (Fig. 1k). Conversely, in the calf, we observed a greater number of capillaries but not arterioles (Fig. 1p,q). Since β1 integrin blockade inhibited both the increase in arterioles in the thigh and capillaries in the calf, β1 integrin function is needed for both arteriogenesis and angiogenesis induced by HI.

### Endothelium-specific knockout of *Itgb1* abrogates arteriogenesis and angiogenesis

**Figure 2:**
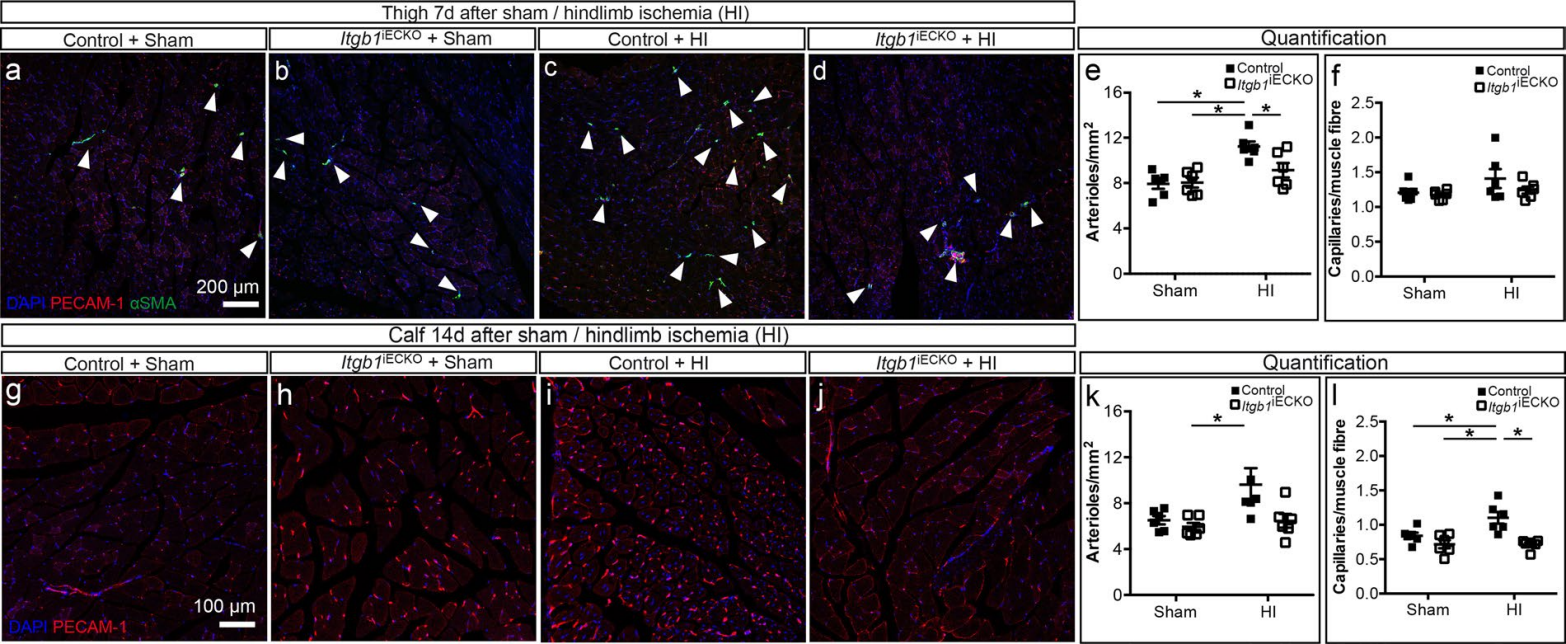
Inhibition of *de novo* arteriogenesis in the thigh and angiogenesis in the calf by endothelium-specific *Itgb1* KO mice following femoral artery ligation. **(a–d)** Fluorescence microscopy images of arterioles and capillaries in the thigh muscle stained for cell nuclei (DAPI, blue), endothelial cells (PECAM-1, red) and smooth muscle cells (α-SMA, green) in **(a)** sham operated *Cre* controls (n = 6), **(b)** sham operated *Itgb1*^iECKO^ mice (n = 6), **(c)** *Cre* controls after 7 days HI (n = 6) and **(d)** *Itgb1*^iECKO^ mice after 7 days HI (n = 6). **(e** +** f)** Quantification of **(e)** arterioles per mm^2^ and **(f)** capillaries per fibre. **(g–j)** Fluorescence microscopy images of capillaries in the calf muscles stained for cell nuclei (DAPI, blue), endothelial cells (PECAM-1, red) in **(g)** sham operated *Cre* controls (n = 6), **(h)** sham operated *Itgb1*^iECKO^ mice (n = 6), **(i)** *Cre* controls after 14 days HI (n = 6), and **(j)** *Itgb1*^iECKO^ mice after 14 days HI (n = 6). **(k** + **l)** Quantification of **(k)** arterioles per mm^2^ and **(l)** capillaries per fibre. *All values are mean* ± standard error of the mean (SEM), statistical significance was determined using two-way ANOVA with Tukey post-hoc test, *p ≤ *0.05*.

To substantiate our findings on β1 integrin, we next conducted HI experiments in endothelium-specific *Itgb1* knockout mice, *Itgb1*^*iECKO*^ (Fig. 2), with the *Cdh5* promoter as an endothelium-specific driver. *Cdh5-CreERT2* mice were used as *Cre* controls, and both mouse lines were treated equally with tamoxifen injections (see Supplementary Fig. S4 for knockout efficiency). Induction of knockout by tamoxifen injection affected neither the number of arterioles and capillaries nor EC viability (Fig. 2, Supplementary Fig. S5 and S6). Notably, *Cre* control mice exhibited *de novo* arteriogenesis (Fig. 2e,k), whereas *Itgb1*^*iECKO*^ mice did not show any increase in the number of arterioles (Fig. 2e,k).

Furthermore, angiogenesis in the calf appeared to be absent in *Itgb1*^*iECKO*^ as indicated by lack of increase in capillary density upon HI (Fig. 2l). Finally, the number of capillaries in the thigh 7d post-HI (Fig. 2f) was unaffected by the endothelial cell-specific deletion of *Itgb1* (Fig. 2f). Taken together, these genetic data and results with β1 integrin blocking antibodies suggest that endothelial β1 integrin is required for both arteriogenesis and angiogenesis after HI.

### Requirement of β1 integrin for flow-mediated dilation (FMD)

**Figure 3:**
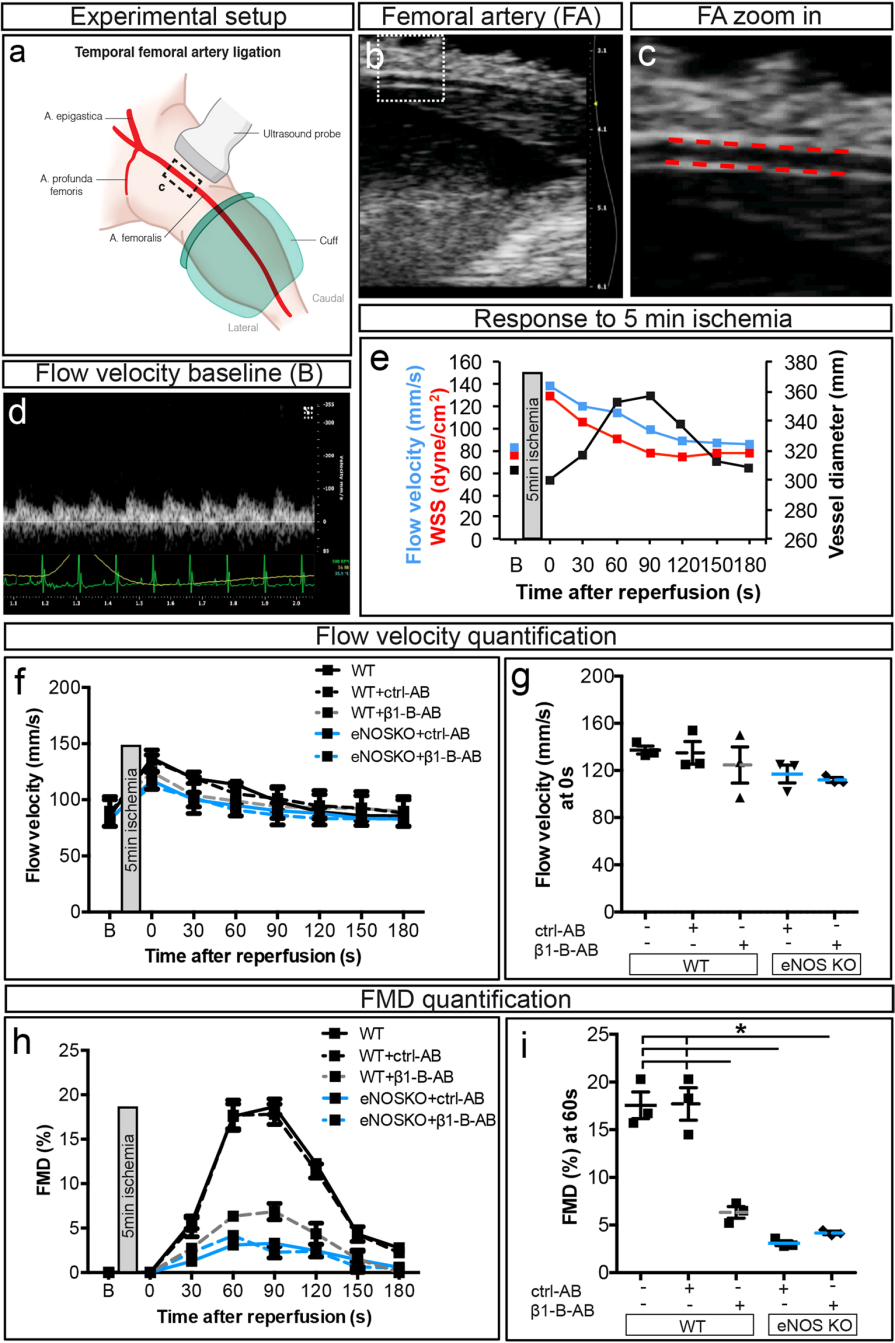
Inhibition of flow-mediated vasodilation in the femoral artery by β1 integrin blocking in mice with and without endothelial nitric oxide synthase (eNOS). **(a)** Experimental setup of flow-mediated dilation (FMD) measurements: see^2^ for details. **(b)** Longitudinal B-mode ultrasound image of FA and **(c)** zoomed image with automatic edge detection. **(d)** Original Doppler flow velocity in FA at baseline. **(e)** Exemplary flow velocity, wall shear stress (WSS), and vessel diameter during reperfusion after response to 5 min ischemia in WT mice. **(f** + **g)** Flow velocity **(f)** over the first 180 s or **(g)** at 0 s during reperfusion after 5 min ischemia in WT (untreated and treated mice with ctrl-AB or β1−Β−ΑΒ) and endothelial nitric oxide synthase (eNOS) knockout (*NOS3*^−/−^) mice (treated with ctrl-AB or β1−Β−ΑΒ) (n = 3, per group). (**h** + **i)** Quantification of FMD **(h)** over the first 180 s or **(j)** at 60 s during reperfusion after 5 min ischemia in WT (untreated, treated with ctrl-AB or β1−Β−ΑΒ) and *NOS3*^−/−^ mice (treated with ctrl-AB or β1−Β−ΑΒ) (n = 3, per group). *All values are mean* ± standard error of the mean (SEM), statistical significance was determined using one-way ANOVA with Tukey post-hoc test, *p ≤ *0.05. No statistical significances are shown in* ***(e)(f)(h)***.

**Figure 4:**
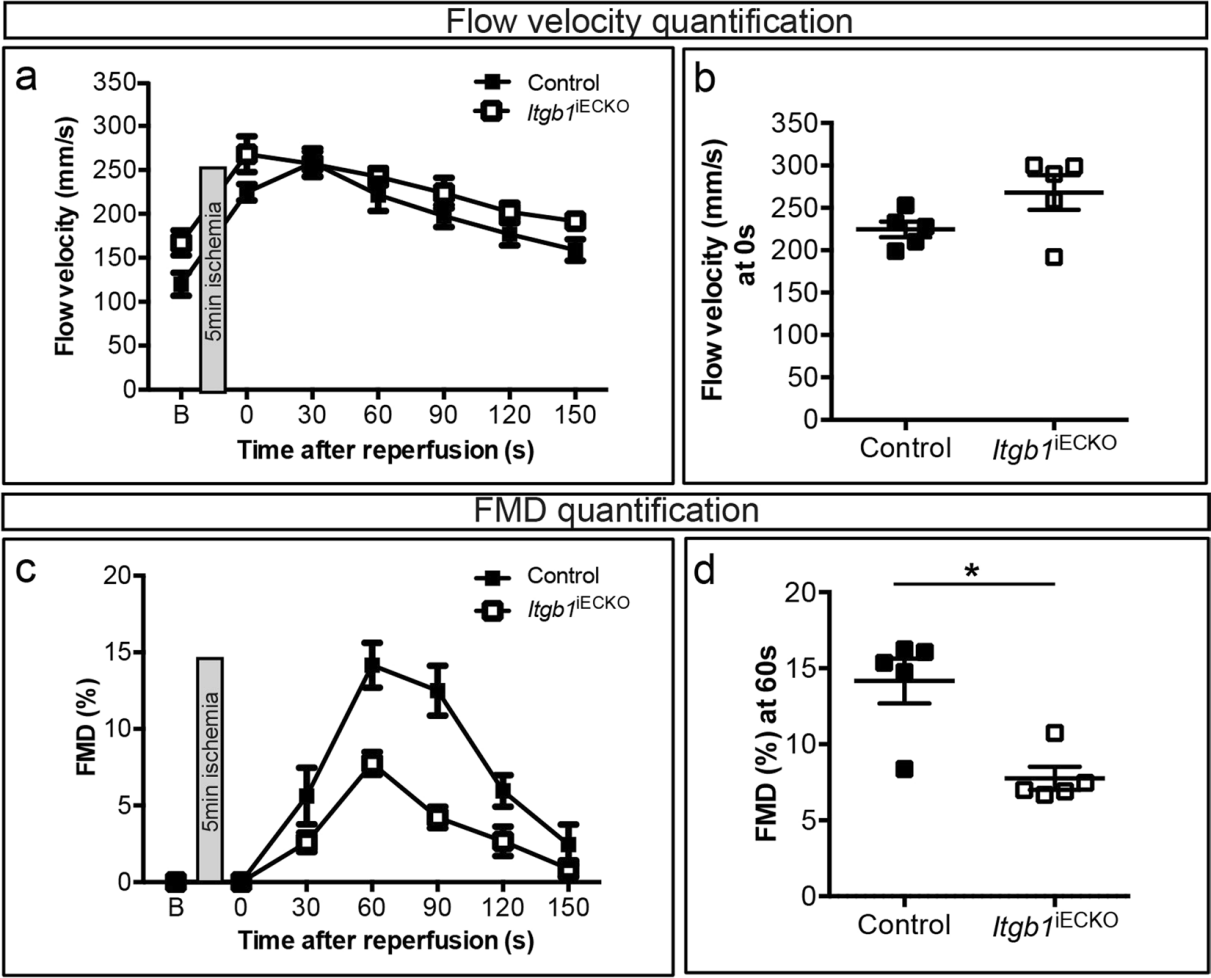
Inhibition of flow-mediated dilation in endothelium-specific *Itgb1* KO mice. **(a** + **b)** Flow velocity **(a)** over the first 150 s or **(b)** at 0 s during reperfusion after 5 min ischemia in *Cre* control mice and *Itgb1*^iECKO^ mice (n = 5, per group). **(c** + **d)** Quantification of FMD over the first 150 s or **(d)** at 60 s during reperfusion after 5 min ischemia in *Cre* control mice and *Itgb1*^iECKO^ mice (n = 5, per group). *All values are mean* ± standard error of the mean (SEM), statistical significance was determined using unpaired two-tailed students t-test, *p ≤ *0.05. No statistical significances are shown in* **(a)(c)**.

To study whether β1 integrin is required for FMD, we measured FMD in conduit arteries of living rodents in a manner that is similar to FMD measurements in humans^2,30^. More specifically in this model, 5 min of lower leg occlusion are used to induce vasodilation of resistance arteries after reperfusion, which in turn increased blood flow and FMD in the upstream FA. Blood flow velocity and FMD were measured in the FA of C57BL/6 J WT mice injected with either β1 integrin blocking antibody or isotype-matched control antibody (Fig. 3; for set-up and basic physiology, see Fig. 3a–e; see Supplementary Fig. S7 for blocking antibody dose-response). The increase in flow velocity in response to acute ischemia remained largely unaffected by either antibody treatment (Fig. 3f,g), indicating that the degree of resistance artery dilation and hence the stimulus for FMD – shear stress – was not substantially affected by the antibodies. Notably, we found that the β1 integrin blockade inhibited FMD, whereas the control antibody had no effect (Fig. 3h,i). In addition, *Itgb1*^*iECKO*^ mice with tamoxifen-induced endothelium-specific *Itgb1* knockout did not differ from *Cre* controls with regard to the flow velocity response to ischemia (Fig. 4a,b). However, *Itgb1*^*iECKO*^ mice exhibited a strongly decreased FMD response (Fig. 4c,d). In summary, the results show that β1 integrin in general and especially in endothelial cells is strictly required for FMD response following an acute HI.

### Potential interaction between β1 integrin and eNOS in flow-mediated dilation (FMD)

To investigate the role of eNOS as a potential downstream effector of β1 integrin (Fig. 3f–i), we performed FMD measurements in mice deficient for *Nos3*, the gene coding for eNOS. The mice were injected with either β1 integrin blocking antibody or isotype-control antibody. While FMD was lower in *Nos3* knockout mice as compared to WT mice, β1 integrin blocking had no further effect on FMD in these *Nos3*-deficient mice. With regard to its subcellular localization, immunohistochemical stainings showed that in EC β1 integrin was not exclusively expressed at the basolateral side, but also present on the luminal side (Supplementary Fig. S8). Finally, knockdown of β1 integrin in human arterial endothelial cells reduced expression levels of total and phosphorylated eNOS (Supplementary Fig. S9 and S10 for uncropped Western blots), further indicating a functional interaction between β1 integrin and eNOS.

## Discussion

Our present data show that endothelial β1 integrin is required for induction of FMD in response to acute increases in blood flow and for arteriogenesis caused by chronically increased blood flow after FA ligation. Our data are based on both antibody blocking and genetic knockout experiments in combination with physiological and morphometric measurements.

In humans, impairments in FMD have been linked to cardiovascular disease development and adverse outcome^31^. While a central role of eNOS is firmly established^31^, the mechanisms of how changes in blood flow are sensed is less clear^4,6^. Our current data show that endothelial β1 integrin is required for induction of FMD in response to acute increases in blood flow. Previous research has already indicated that integrins are involved in vasodilation in experimental models (i.e., murine mesenteric resistance arteries and porcine coronary arterioles)^32,33^. For instance, in isolated mesenteric resistance arteries *in vitro* α1 integrin played an important role in FMD through activation of phosphoinositide 3-kinase (PI3K) and Akt serine/threonine kinases^32^. In isolated porcine arterioles, it was shown that activation of endothelial αvβ3 and α5β1 integrins mediated coronary arteriolar dilation via the endothelial production of cyclooxygenase-derived prostaglandins^34^, a pathway that seems to be less relevant in peripheral artery FMD in humans and rodents^2,30^. In another study, also using isolated porcine arterioles, it was observed that abluminally located β3 integrin was involved in FMD using a blocking antibody while it was unaffected by a macromolecular complex of inhibitors that remained intraluminal^33^. In contrast, a more recent *in vitro* study on bovine aortic endothelial cells suggested that apically expressed β1 integrin was rapidly activated in response to acute shear stress^28^. In these EC, the blockade of β1 integrin activation attenuated a shear-induced signalling cascade involving Src-family kinase, PI3K, Akt, and eNOS on the apical endothelial cell surface^27^. Furthermore, Xanthis *et al*. recently showed that β1 integrin was a key sensor of unidirectional shearing forces using *in vitro* flow systems and magnetic tweezers on apical β1 integrin in human umbilical vein endothelial cells (HUVEC)^35^. The authors also observed that β1 integrin knockout inhibited both acute Ca^2+^ responses and eNOS expression increases due to shear stress. Using super-resolution microscopy, they showed that activation of luminal/apical β1 integrin but not basolateral β1 integrin in mouse aorta endothelial cells correlated with shear responses *in vivo*. While these data show that apical β1 integrin can respond to force, they do not preclude the important and well-established role for basally located integrins. Our current data support that β1 integrin is expressed at the apical and basolateral sides in ECs. As both sides may contribute to flow sensing, the mechanisms how endothelial cells integrate microenvironmental downstream signals from apical and basal β1 integrin need to be further investigated^35^. To date, due to a previous lack of a methodology to measure FMD *in vivo* in mice^2^, the role of endothelial β1 integrin in clinically relevant FMD have not yet been investigated. Our data show that FMD is significantly and dose-dependently inhibited by β1 integrin blocking antibodies and endothelial specific knockout of β1 integrin. Furthermore, we show that this effect was similar to *NOS3* knockout animals in which β1 integrin blocking did not further inhibit FMD. Furthermore, our *in vitro* data indicate that β1 integrin contributes to eNOS expression and activation.

As a response to ischemia, changes in blood flow lead to acute vasodilation and chronically to vascular remodelling processes, such as angiogenesis and arteriogenesis. While it was previously shown that β1 integrin was upregulated in collaterals during arteriogenesis^27^, it was unknown if β1 integrin was required for collateral growth in the hindlimb. Others have shown using α5 integrin-targeted microbubbles in a murine iliac occlusion hindlimb model that a signal enhancement from α5 integrin coincides with early blood flow recovery in the adductor muscles^36^. Furthermore, β1 integrin expression was shown to be upregulated in response to hypoxia via activation of the transcription factor HIF-1α in fibroblasts during wound healing^29^. Our current data integrate with these previous findings and provide evidence for the essential role of endothelial β1 integrin during HI-induced arteriogenesis in mice. Notably, endothelium-specific deletion of the integrin does not induce EC-apoptosis, indicating that the observed effects are not due to changed EC-viability in general. However, further work is needed to uncover the full molecular mechanisms by which β1 integrin in EC contributes to FMD, arteriogenesis, and angiogenesis. Detection of differences in formation of heterodimers, activity, or post-translational modifications may help to explain the responses in EC and the role of hypoxia in this context. Furthermore, eNOS may play a critical role in β1 integrin-mediated regulation of FMD as well as in arteriogenesis and angiogenesis. Our current data show that β1 integrin knockdown decreases total and phosphorylated eNOS *in vitro* suggesting a potential functional interaction. Previous literature showing that eNOS knockout mice have a phenotype with impaired FMD, arteriogenesis, and angiogenesis^13,14^ similar to the one of the mice we generated for the experiments described here. In face of the peripheral artery disease epidemic worldwide^37^ with essentially no specific pharmaceutical therapies available at hand^38^, a better understanding of the mechanisms underlying compensatory vascular maintenance and regeneration is essential to develop therapies in the future.

We conclude that β1 integrin is required for acute and chronic endothelium-dependent adaptations of the vasculature to HI. Furthermore, β1 integrin might act in concert with eNOS to induce FMD and is needed in vascular endothelial cells for arteriogenesis and angiogenesis following a permanent, chronic occlusion of the FA. Therefore, β1 integrin-mediated signalling events provide a framework for better understanding acute and chronic adaptation of the arterial vasculature to ischemia in the hindlimb.

## Methods

We have performed 4 sets of experiments (see Supplementary Fig. S1 for overview of study groups and protocols). *Study 1*: In wild type C57BL/6 J mice (WT), we compared collateral expansion, *de novo* arteriogenesis, and angiogenesis in the thigh and calf after HI by FA ligation with sham operated animals. To investigate the role of β1 integrin, we injected either a β1 integrin blocking antibody or control antibody in HI. *Study 2*: To study the role of endothelial β1 integrin, we compared arteriogenesis and angiogenesis (HI vs. sham) in mice with conditional endothelial cell specific deletion of β1 integrin (*Itgb1*^iECKO^) with Cre control mice (Control). *Study 3*: To investigate the role of β1 integrin and eNOS in acute flow-dependent tone regulation of hindlimb conduit arteries, we compared microvascular flow velocity response and FMD in untreated WT mice with WT and eNOS knockout (NOS3^−/−^) animals that had either received a β1 integrin blocking antibody or isotype-control antibody. *Study 4*: To study the role of endothelial β1 integrin in acute arterial flow-dependent tone regulation, we compared microvascular flow velocity response and FMD in mice with conditional endothelial cell specific deletion of β1 integrin (*Itgb1*^iECKO^) with Cre control mice (Control).

### Mice

For all experiments 10 to 12 week old C57BL/6 J (Janvier) mice were used as well as global *NOS3* knockouts^39^. Furthermore, for conditional endothelial cell specific deletion of *Itgb1*, *Cdh5-CreERT2* mice^40,41^ were crossed with *Itgb1*-loxP mice (*Itgb1*^iECKO^)^42^. *Cdh5-CreERT2* mice were used as controls with the same amount and frequency of tamoxifen injections. Knockout and control mice were intraperitoneally injected with 100 µl tamoxifen solution (75 mg/kg bw) for 5 days. All experiments were performed according to the German animal protection laws and the experimental protocols (project numbers: 84-02.04.2012.A404, 84-02.04.2014.A312, 84-02.04.2015.A241) were approved by the institutional licensing committee (Animal Ethics Committee of the Landesamt für Natur, Umwelt und Verbraucherschutz, North-Rhine-Westphalia, Germany).

### Hindlimb ischemia protocol

The mice were anesthetized with ketamine (100 mg/kg) and xylazine (10 mg/kg) intraperitoneal. The left FA was prepared carefully without damaging the vein and nerve and was ligated distal to the origin of the arteria profunda femoris with two sutures (Prolene 5/0, Ethicon). Between the two sutures the artery was cut. The wound was closed, and the animals were allowed to recover.

### Laser-Doppler-Imaging for perfusion analysis

Before, immediately after or 3d post HI perfusion in thigh and calf were analysed^45^. Therefore, mice were anesthetized with 2.5–3.0 vol.-% isoflurane induction and 1.5 vol.-% maintenance and placed on a 37 °C heating pad. For imaging both hindlimbs, operated and control one were scanned and perfusion rates were calculated on the basis of a color-coded histogram pixel.

### Near infrared spectroscopy

For determination of oxygen levels in thigh and calf images with a near infrared spectroscopy (NIRS, Kent imaging), mice hindlimbs were imaged before, immediately after, and 7d post HI.

### Injection of β1 integrin blocking antibody

The β1 integrin blocking antibody and the control antibody were injected intravenously two times per week at a concentration of 1 mg/ml (100 µl) into the mice starting preoperatively. The injections were performed in random order by a different person so that investigators were blinded to the treatment regime. The β1 integrin blocking antibody was a purified NA/LE hamster anti-rat CD29 (555002, BD Bioscience). A purified Armenian Hamster IgM Isotype control antibody (401006, Biolegend) was used as a control.

### Immunostaining and cell death detection via TUNEL

On day 7 and 14 after surgery, mice were anesthetized with ketamine (100 mg/kg) and xylazine (10 mg/kg). Mice were euthanized and the left ventricle of the heart was cannulated and perfused for 2 minutes at 100 mmHg with Ringer solution containing 0.9% adenosine, 0.1% sodium nitroprusside, and 0.05% BSA (wt/vol) at 37 °C followed by 4% paraformaldehyde. The thigh and calf muscles were dissected and immersed in 15% o.n. and 30% sucrose before cryo embedding.

Histological analyses were performed on transversal sections (12 µm) of the thigh and the calf of each mouse and fixed in 4% paraformaldehyde. Slides were blocked and stained with primary antibodies for rat anti-PECAM-1 antibody (550274, BD Bioscience) at 1/50 dilution or goat anti-PECAM-1 antibody (AF3628, R&D) at 1/20 dilution, primary mouse anti-SMA-Cy3 (C6198, Sigma Aldrich) at 1/100 dilution, primary rat anti-β1 integrin antibody (MAB1997, Millipore) at 1/200 dilution and primary rat anti-IgG2b (isotype-ctrl) antibody (ab18541, Abcam) at 1/100 dilution and incubated for 1 h at room temperature (RT) or over night at 4 °C. Slides were then washed and secondary antibody conjugated with Alexa Fluor 488 (A32814 or A-21208, ThermoFischer Scientific), Cy3 (712-165-153, Jackson ImmunoResearch) and Cy5 (712-175-153, Jackson ImmunoResearch) was added at 1/500 dilution and incubated in the dark at RT for 1 h. Slides were mounted using Vectashield-DAPI mounting medium (Vector Laboratories) or DAPI was diluted 1/1,000 in secondary antibody incubation step. Random microscopic images from 2–3 different sections in each tissue block were examined for the presence of arterioles and capillaries. Pictures were acquired with a Nikon Eclipse Ti-S confocal microscope (Nikon), with an Axio Vert LSM710 confocal laser scan microscope (Zeiss) or Leica TCS SP8 STED3X (Leica). The capillary density was assessed relative to the number of muscle fibres and the arterioles numbers were calculated to area in mm^2^ using FIJI (Image J, National Institutes of Health). Apoptotic endothelial cells were detected via an *in situ* cell death detection kit (TUNEL) according to the manufacture’s instruction (12156792910, Roche). Co-staining of endothelial cells (PECAM-1) and cell nuclei (DAPI) was performed as described above.

### LacZ staining

For analysis of KO efficiency, thighs from *Itgb1*^iECKO^ and control mice were isolated and incubated in 2% PFA over night at 4 °C. Thereafter, the tissue was stained in staining solution (5 mM potassium ferricyanide, 5 mM potassium ferrocyanide, 2 mM magnesium chloride, 0.01% sodium deoxycholate and 0.02% Nonidet P-40 diluted in PBS, pH 7.2–7.4). Directly before use, 25 μl of a 40 mg/ml X-Gal (5-brom-4-chloro-3-indolyl β-D-galactopyranoside, B4252, Sigma Aldrich, diluted in dimethyl-formamide) stock solution was added to 1ml of staining solution. Thighs of *Itgb1*i^ECKO^ and control mice were incubated in staining solution over night at 37°C before imaging.

### Visualization of collaterals using PU4 resin

On day 7 after FA ligation, mice were anesthetized with ketamine (100 mg/kg) and xylazine (10 mg/kg). Mice were euthanized and the aorta was cannulated and perfused for 2 min at 100 mmHg with Ringer solution containing 0.9% adenosine, 0.1% sodium nitroprusside, and 0.05% BSA (wt/vol) at 37 °C. Afterwards PU4ii resin and hardener (Vasqtec) were mixed and quickly injected. The hindlimbs were digested with 1% Natriumhydroxide (AppliChem) and images were taken with a stereo microscope (Nikon SMZ1500). Measurements were performed by an observer blinded to sample allocation. The collateral arteries were measured at maximally 15 different random locations in every mouse.

### Measurement of flow-mediated dilation in mice

The protocol was previously published^2^: Briefly, mice were anesthetized with isoflurane and the fur was removed from the hindlimbs. Then the animals were transferred to a warmed ultrasound investigation table equipped with ECG. A vascular occluder (5 mm diameter, Harvard Apparatus) was placed around the lower limb to induce occlusion of the distal hindlimb as an ischemic trigger. Pre-warmed ultrasound gel was applied to the proximal inner thigh, the ultrasound probe was manually aligned with visible blood vessels, and arterial blood flow in the FA confirmed by pulsed wave (PW) Doppler. The images of the FA were optimized to achieve clear contrast of the vessel walls. After recording baseline readings, the vascular occlude was inflated manually with an air-filled syringe. Following 5 min of HI, the cuff was deflated and FA diameter and blood flow velocity measurements were recorded for 180 s at 30 s intervals. The recorded loops were analysed off-line by an operator blinded to group allocation using a semi-automated system (Brachial Analyzer, MIA) which is also used for human FMD analyses as described by Heiss *et al*.^30^. FMD was determined as ∆% in average FA diameter following reperfusion as compared to baseline pre-ischemic values: [(Diameter_post-ischemic_ – Diameter_baseline_) ⁄ Diameter_baseline_] * 100. All diameter readings were taken at end diastole.

To study the role of β1 integrin as a central mechanisms initiating FMD in mice (10–12 week old male), FMD was measured after β1 integrin blocking or control antibody injection (concentration described before) intravenously in C57BL/6 J and age-matched eNOS knockout mice^39^. For dose-dependence analysis β1 integrin blocking antibodies were diluted 1/1,000 or 1/100. Furthermore, we tested FMD in mice with conditional endothelial cell specific deletion of *Itgb1*. Therefore, *Cdh5-CreERT2* mice were crossed with *Itgb1*-loxP mice and results were compared to tamoxifen-injected *Cdh5-CreERT2* control mice.

### Magnetic-Activated Cell Sorting of endothelial cells and quantitative real-time PCR

To isolate EC from *Itgb1*^iECKO^ and control mice hindlimbs, magnetic-activated cell sorting (MACS) was performed. Therefore, hindlimbs were dissociated with a gentleMACS Dissociator (Milteny Biotech). According to the manufacture protocol a single-cell suspension was prepared and CD45 positive cells were depleted by labelling with ‘CD45 MicroBeads’ (130-052-301, Milteny Biotech). CD45 positive cells, as leukocyte subtypes and platelets were depleted as they express PECAM-1 (CD31) as well, which was used in the next step for positive selection of EC. Following separation in magnetic field cell solution was labelled with ‘CD31 MicroBeads’ (130-097-418, Milteny Biotech) to finally isolate EC from hindlimbs.

To analyse β1 integrin expression in EC, cell pellets from MACS procedure were suspended in peqGold TriFast (PEQLAB) and total RNA was isolated by phenol/chloroform extraction^43^. RNA was transcribed in cDNA with SuperScript® II Reverse Transcriptase (Invitogen). Quantitative real-time PCR was completed by using Brilliant III Ultra-Fast SYBR® Green QPCR Master Mix and the thermal cycler Stratagene Mx3000P (Agilent Technologies). Samples were analyzed in duplicates. Primer sequences as followed:

mouse *itgb1* forward: 5′-AATGCCAAGTGGGACACGGG-3′

mouse *itgb1* reverse: 5′-TGACTAAGATGCTGCTGCTGTGAGC-3′

mouse *β2m* forward: 5′- GAGCCCAAGACCGTCTACTG-3′

mouse *β2m* reverse: 5′-GCTATTTCTTTCTGCGTGCAT-3′

Expression of endothelial β1 integrin was determined by means of 2^-ΔΔCt-value^44^ and therefore, *β2m* was used as housekeeping gene.

### Primary human cell culture and transfection

Primary human coronary artery endothelial cells (HCAEC, male, 21 years old), purchased from PELOBiotech, were cultured in a humidified atmosphere at 5% CO_2_ and 37 °C using microvascular endothelial cell growth medium kit enhanced (PELOBiotech). HCAEC were grown up to passage 6. Cell culture dishes were pre-coated with speed coating solution (PELOBiotech). For knocking down the expression of β1 integrin, HCAEC were transfected with 250 nM *ITGB1*-siRNA (5′-CCUAAGUCAGCAGUAGGAACAUUAU-3′, Invitrogen) or a non-targeting control siRNA (Invitrogen) with a similar GC-content by electroporation (4D-NucleofectorTM System, LONZA) and incubated for 48 h. eNOS expression, phosphorylation, and β1 integrin knockdown efficiencies were analysed by Western blotting.

### Western blotting

Cultured and transfected HCAEC were lysed in RIPA buffer (50 mM Tris/HCl pH 7.4, 150 mM NaCl, 1% IGEPAL, 0.25% Na-deoxycholate, 1 mM EDTA) with protease inhibitors (cOmplete Protease Inhibitor Cocktail Tablets, Roche) and phosphatase inhibitors (Phosphatase Inhibitor Cocktail, Roche). After lysis, fresh samples were directly used for protein analysis. The lysates were heated in Laemmli buffer (Bio-Rad) containing β-mercaptoethanol (Roth) at 95 °C for 5 min. For SDS-PAGE, 4–15% SDS-gels (Bio-Rad) and Mini-PROTEAN Tetra Cell system (Bio-Rad) were used. The proteins were transferred to a PVDF membrane by means of the Trans-Blot Turbo Transfer system (Bio-Rad). The membrane was incubated with rabbit anti-GAPDH (Abcam, ab9485) at 1/5,000 dilution, rabbit anti-eNOS (9572S, Cell Signaling Technologies) at 1/500 dilution, rabbit anti-Phospho(Ser1177)-eNOS (9571S, Cell Signaling Technologies) at 1/500 dilution or goat anti-β1 integrin (Santa Cruz, sc-6622) at 1/2,000 dilution as primary antibodies overnight at 4 °C, and HRP-conjugated donkey anti-rabbit (7074, Cell Signaling Technologies) at 1/2,000 dilution and donkey anti-goat (705-035-147, Jackson ImmunoResearch) at 1/5,000 dilution as secondary antibodies for 1 h at RT. Chemiluminescence was detected using WesternBright Quantum Kit (Advansta) and ChemiDoc MP Imaging System (Bio-Rad). For expression and phosphorylation evaluation, semi-quantitative band density analysis was performed in FIJI (ImageJ NIH), where GAPDH was used as housekeeping protein for normalization.

### Statistical analyses

Statistical analyses were performed with Prism 6.0 for Mac OS X (GraphPad Software Inc.). All data are presented as mean ± standard error of the mean. Group differences were calculated with one- or two-way ANOVA and consecutive post hoc test (Tukey) or t-test if only 2 groups were compared. P-values less than or equal to 0.05 were regarded as significant.

## Supplementary information

Supplementary Figures
