# Supplementary material for: Requirement of β1 integrin for endothelium-dependent vasodilation and collateral formation in hindlimb ischemia
Source: Sci Rep. 2019 Nov 15;9:16931. doi: 10.1038/s41598-019-53137-x (PMC6858366; doi:10.1038/s41598-019-53137-x)
Supplement: Supplementary file 1 — Supplementary Figures [file 41598_2019_53137_MOESM1_ESM.pdf]

Requirement of β1 integrin for endothelium-dependent vasodilation and collateral formation in hindlimb ischemia

Carina Henning[1,2], Anna Branopolski[1,2*], Dominik Schuler[2*], Dimitrios Dimitroulis[2], Patrik Huelsemann[2], Christopher Nicolaus[2], Roberto Sansone[2], Jelle Ludolf Postma[3], Daniel Eberhard[1], Ferdinand Le Noble[4], Malte Kelm[2], Eckhard Lammert[1,5*], Christian Heiss[2,6,7*]*

[1]Institute of Metabolic Physiology, Heinrich Heine University Duesseldorf, Germany,[2]Division of Cardiology, Pulmonology, and Vascular Medicine, Medical Faculty, University Duesseldorf, Germany, [3]Center for Advanced Imaging, Heinrich Heine University Duesseldorf, Germany, [4]Institute for Zoology, Karlsruhe Institute of Technology, Karlsruhe, Germany, [5]Institute for Vascular and Islet Cell Biology, German Diabetes Center (DDZ) - Leibniz Center for Diabetes Research, Duesseldorf, Germany

Author's changed current affiliation:
[6]University of Surrey, Faculty of Health and Medical Science, Guildford, United Kingdom, [7]Surrey and Sussex NHS Healthcare Trust, Redhill, United Kingdom

*equal contribution

Address for correspondence:
Prof. Dr. med. Christian Heiss (ORCID: 0000-0002-3212-8995)
University of Surrey, Faculty of Health and Medical Science
Stag Hill
Guildford GU2 7XH, United Kingdom
Tel: +44 7878589817, Email: c.heiss@surrey.ac.uk

Prof. Dr. rer. nat. Eckhard Lammert
Institute of Metabolic Physiology
Heinrich-Heine-University Duesseldorf
Universitaetsstr. 1
Germany
Tel.: +49-211-811-4990, Fax: +49-211-811-3897
Email: lammert@hhu.de

SUPPLEMENTARY FIGURE LEGENDS

Supplementary Fig. S1

Summary of individual study protocols.

(a) *S*tudy 1: Wild type C57BL/6J mice (WT), were analyzed for classical arteriogenesis, *de novo* arteriogenesis, and angiogenesis in the thigh and calf after femoral artery (FA) ligation (introducing hindlimb ischemia, HI) versus Sham operation. To investigate the role of β1 integrin, β1-B-AB or ctrl-AB were injected. (OP=operation to induce HI).

(b) Study 2: Mice with conditional endothelial cell-specific deletion of *Itgb1* (*Itgb1*^{iECKO}) versus *Cdh5-CreERT* controls were studied to investigate the role of endothelial β1 integrin in *de novo* arteriogenesis and angiogenesis (HI vs Sham).

(c) Study 3: To investigate the role of β1 integrin and eNOS in acute flow-dependent tone regulation of hindlimb conduit arteries, microvascular flow response and flow-mediated dilation (FMD) were compared in WT and eNOS knockout (*NOS3*$^{-/-}$) animals treated with no AB, β1-B-AB or ctrl-AB.

(d) Study 4: To study the role of endothelial β1 integrin in acute arterial flow-dependent tone regulation, mice with conditional endothelial cell-specific deletion of *Itgb1* versus *Cdh5-CreERT* control mice were compared in terms of microvascular flow response and FMD.

Supplementary Fig. S2

Perfusion and oxygenation in thigh and calf upon femoral artery ligation.

Laser Doppler imaging for analysis of perfusion and near infrared spectroscopy (NIRS) for assessment of muscle oxygenation after HI. **(a-c)** Representative Laser Doppler images of legs **(a)** before, **(b)** immediately after, and **(c)** 3 days after HI introduced by FA ligation compared to their respective control legs without HI. **(d-f)** Quantification of the perfusion, shown as % of control leg, in the thigh and calf **(d)** before, **(e)** immediately after, and **(f)** 3 days after FA ligation. **(g-i)** Representative NIRS images of legs **(g)** before, **(h)** immediately after, and **(i)** 7 days after FA ligation. *All values are mean ± standard error of the mean (SEM), statistical significance was determined using paired, two-tailed Student's t-test,* $^{*}p\leq 0.05$

Supplementary Fig. S3

Change in endothelial β1 integrin expression in thigh and calf following femoral artery ligation.

Analysis of endothelial β1 integrin expression in the thigh and calf muscles at 7 days post-HI. **(a-d)** Representative LSM images of vessels in the thigh muscle stained for cell nuclei (DAPI, blue), EC (PECAM-1, red) and β1 integrin (green) in **(a+b)** Sham operated leg and **(c+d)** WT mice after 7 days HI. **(e)** Quantification of endothelial β1 integrin expression, represented by the mean intensity of β1 integrin within PECAM-1 positive areas, shown as % of Sham leg (n=3 each). **(f-i)** Representative LSM images of vessels in the calf muscle stained for cell nuclei (DAPI, blue), EC (PECAM-1, red) and β1 integrin (green) in **(f+g)** Sham operated and **(h+i)** WT mice after 7 days HI. **(j)** Quantification of endothelial β1 integrin expression, represented by the mean intensity of β1 integrin within PECAM-1 positive areas (n=3 each). *All values are mean ± standard error of the mean (SEM), statistical significance was determined using paired, two-tailed Student's t-test,* $^{*}p\leq 0.05$

Supplementary Fig. S4

Recombination and KO efficiency of tamoxifen-induced endothelial cell-specific *Itgb1* deletion in adult mouse hindlimbs. (a-f) Analysis of tamoxifen-induced endothelial *Itgb1* recombination. Images of LacZ staining of the thigh muscle of **(a-c)** three tamoxifen-induced *Cdh5-CreERT* control mice (Cre controls) and **(d-f)** three *Itgb1*^{iECKO} mice. Arrows are exemplary indicating blood vessels within the skeletal muscle. **(g)** Quantification of *Itgb1* gene expression in EC isolated from Cre control (n=8) and *Itgb1*^{iECKO} (n=8) hindlimbs using *β2m* as housekeeping gene. *All values are mean ± standard error of the mean (SEM), statistical significance was determined using unpaired, two-tailed Student's t-test, * p≤0.05*

Supplementary Fig. S5

Impact of β1 integrin knockout induction on number of arterioles and capillaries in thigh and calf.

(a-d) Quantification of **(a+c)** arteriole numbers per mm^2 and **(b+d)** capillaries per muscle fibre in **(a+b)** thigh and **(c+d)** calf before (pre) (n=4) and after (post) (n=6) knockout induction by tamoxifen injections. *All values are mean ± standard error of the mean (SEM), statistical significance was determined using unpaired, two-tailed Student's t-test, * p≤0.05.*

Supplementary Fig. S6

EC apoptosis in endothelium-specific β1 integrin knockout versus control mice.

(a-d) Representative LSM images of the thigh muscle in tamoxifen-induced *Cdh5-CreERT* control mice (Control) stained for cell nuclei (DAPI, blue), EC (PECAM-1, red) and apoptotic cells (TUNEL, green). **(e-h)** Representative LSM images of the thigh muscle in tamoxifen-induced *Itgb1*^{iECKO} mice stained for cell nuclei (DAPI, blue), EC (PECAM-1, red) and apoptotic cells (TUNEL, green). Arrows are exemplary indicating the TUNEL positive EC in the muscle. **(i)** Quantification of TUNEL positive EC related to the total EC number in hindlimbs of control and *Itgb1*^{iECKO} mice. *All values are mean ± standard error of the mean (SEM), statistical significance was determined using unpaired, two-tailed Student's t-test, * p≤0.05.*

Supplementary Fig. S7

Dose-dependent inhibitory effect of β1 integrin blocking antibody on flow-mediated dilation (FMD).

(a) Quantification of FMD over the first 180 s or **(b)** at 60 s during reperfusion after 5 min ischemia in WT mice (n=3-5) treated with ctrl-AB or β1-B-AB at 1/1 (originally applied concentration), 1/100- or 1/1,000-diluted concentration. *All values are mean ± standard error of the mean (SEM), statistical significance was determined using one-way ANOVA with Tukey post-hoc test, * p≤0.05. No statistical significances are shown in **(a)**.*

Supplementary Fig. S8

Subcellular localization of endothelial β1 integrin.

Immunohistochemical assessment of subcellular localization of endothelial β1 integrin by using two adjacent sections. **(a-h)** Representative LSM images of an artery in the thigh stained for cell nuclei with DAPI (blue), EC (PECAM-1, red), **(a-d)** β1 integrin antibody (green) or **(e-h)** isotype ctrl-antibody (green).

Supplementary Fig. S9

Effect of β1 integrin knockdown on eNOS phosphorylation (Ser1177) in human arterial endothelial cells *in vitro*.

(a-c) Semi-quantitative analysis of **(b)** eNOS phosphorylation (Ser117) and **(c)** eNOS protein expression related to GAPDH protein expression as housekeeping protein in human coronary artery endothelial cells (HCAEC) treated with control siRNA (ctrl-siRNA) (n=4) and *ITGB1*-siRNA (β1-siRNA) (n=4). **(d-e)** Semi-quantitative analysis of **(e)** β1 integrin protein expression related to GAPDH protein expression as housekeeping protein in HCAEC treated with ctrl-siRNA (n=3) and β1-siRNA (n=3).
*All values are mean ± standard error of the mean (SEM), statistical significance was determined using unpaired, two-tailed Student's t-test, * $p \leq 0.05$.*

Supplementary Fig. S10
Uncropped Western blots for eNOS, phosphorylated eNOS, and β1 integrin upon *ITGB1* knockdown in human coronary arterial endothelial cells *in vitro*.

(a) Uncropped Western blot from Supplementary Fig. 9 (a), (left side) presenting GAPDH, short exposure and (right side) P(S1177)-eNOS and eNOS, long exposure in ctrl-siRNA and β1-siRNA treated HCAEC. **(b)** Uncropped Western blot from Supplementary Fig. 9 (d), presenting GAPDH and β1 integrin in ctrl-siRNA and β1-siRNA treated HCAEC.

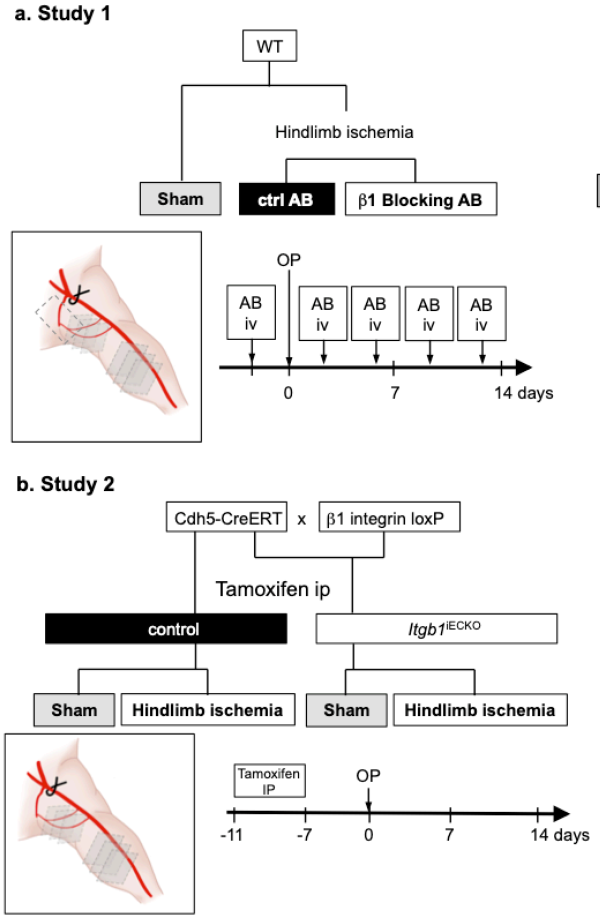

Supplementary Fig. S1

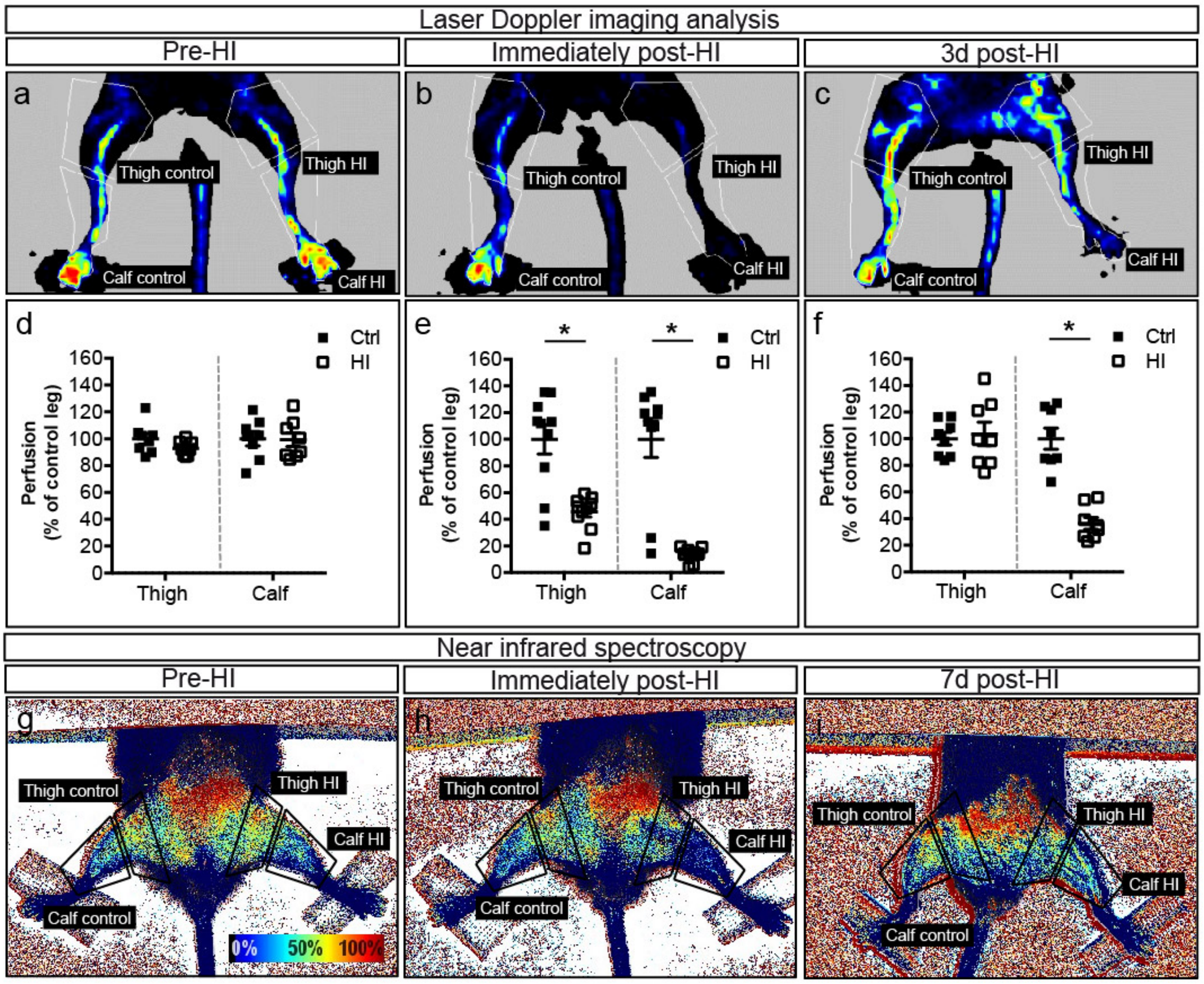

Supplementary Fig. S2

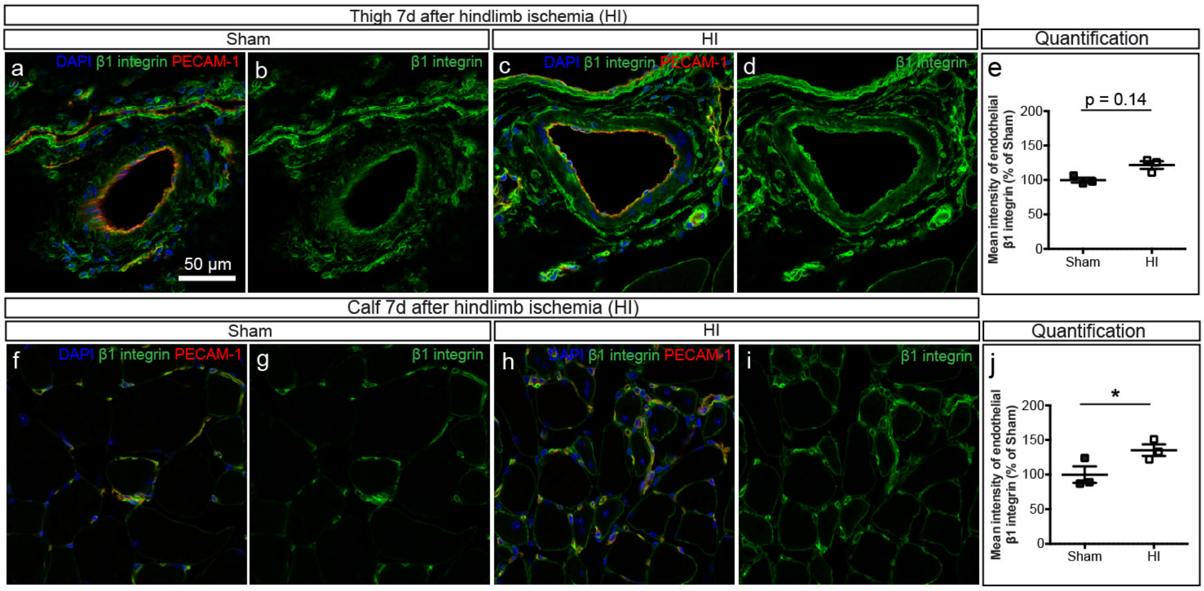

Supplementary Fig. S3

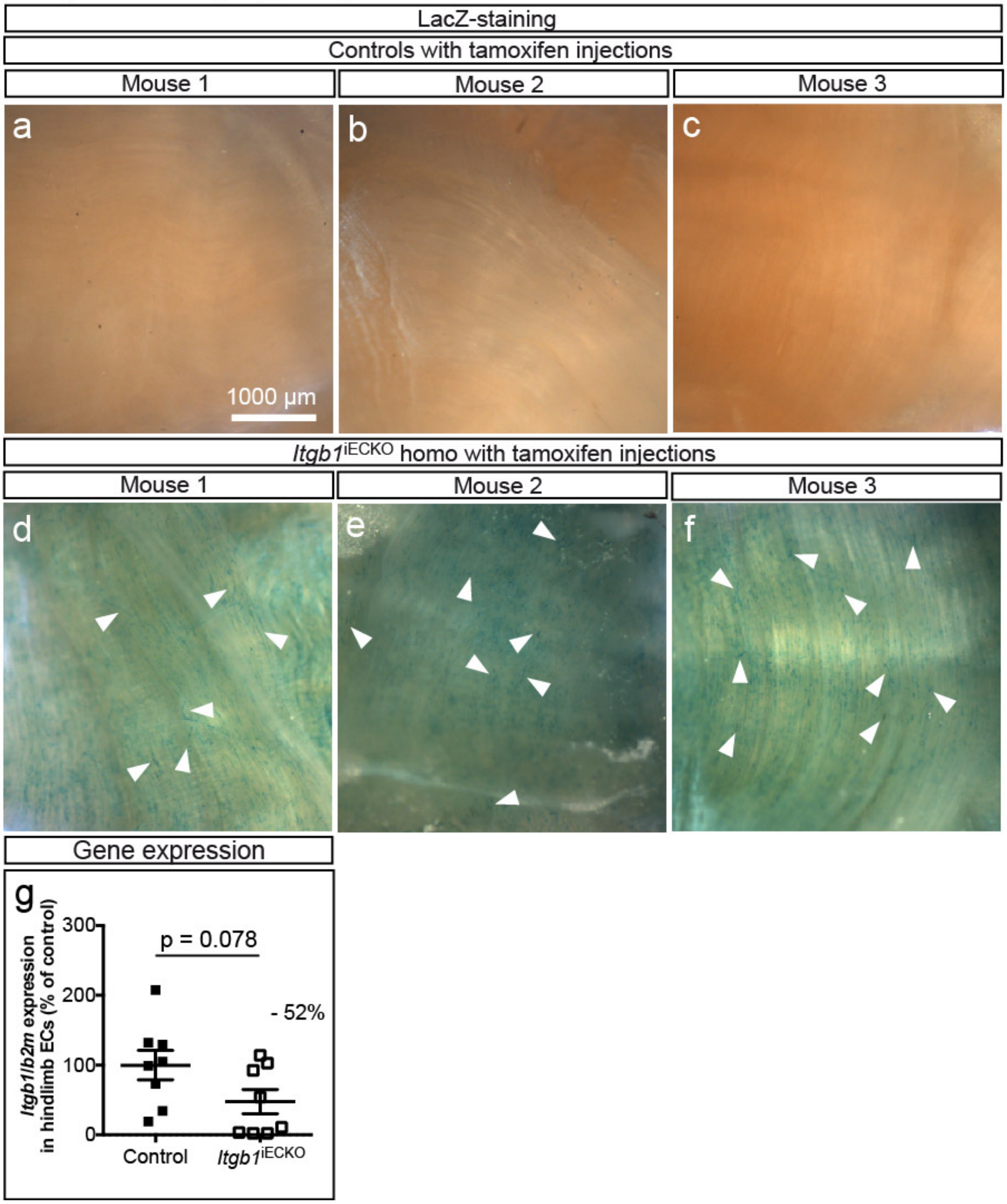

Supplementary Fig. S4

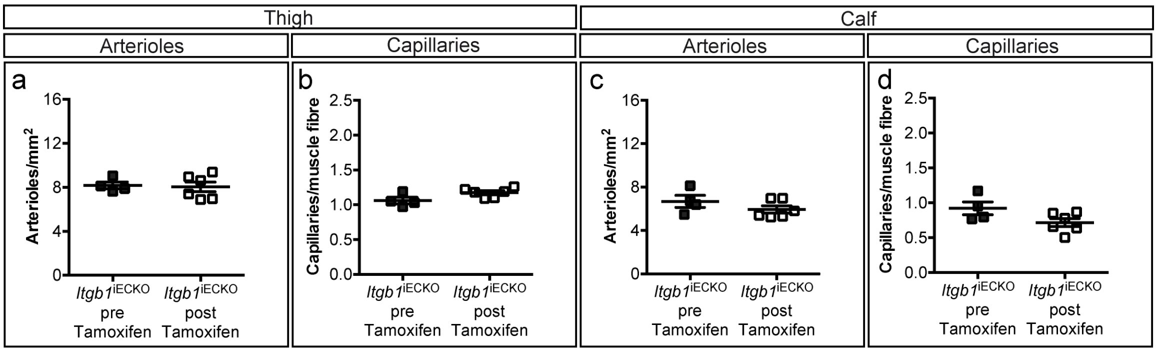

Supplementary Fig. S5

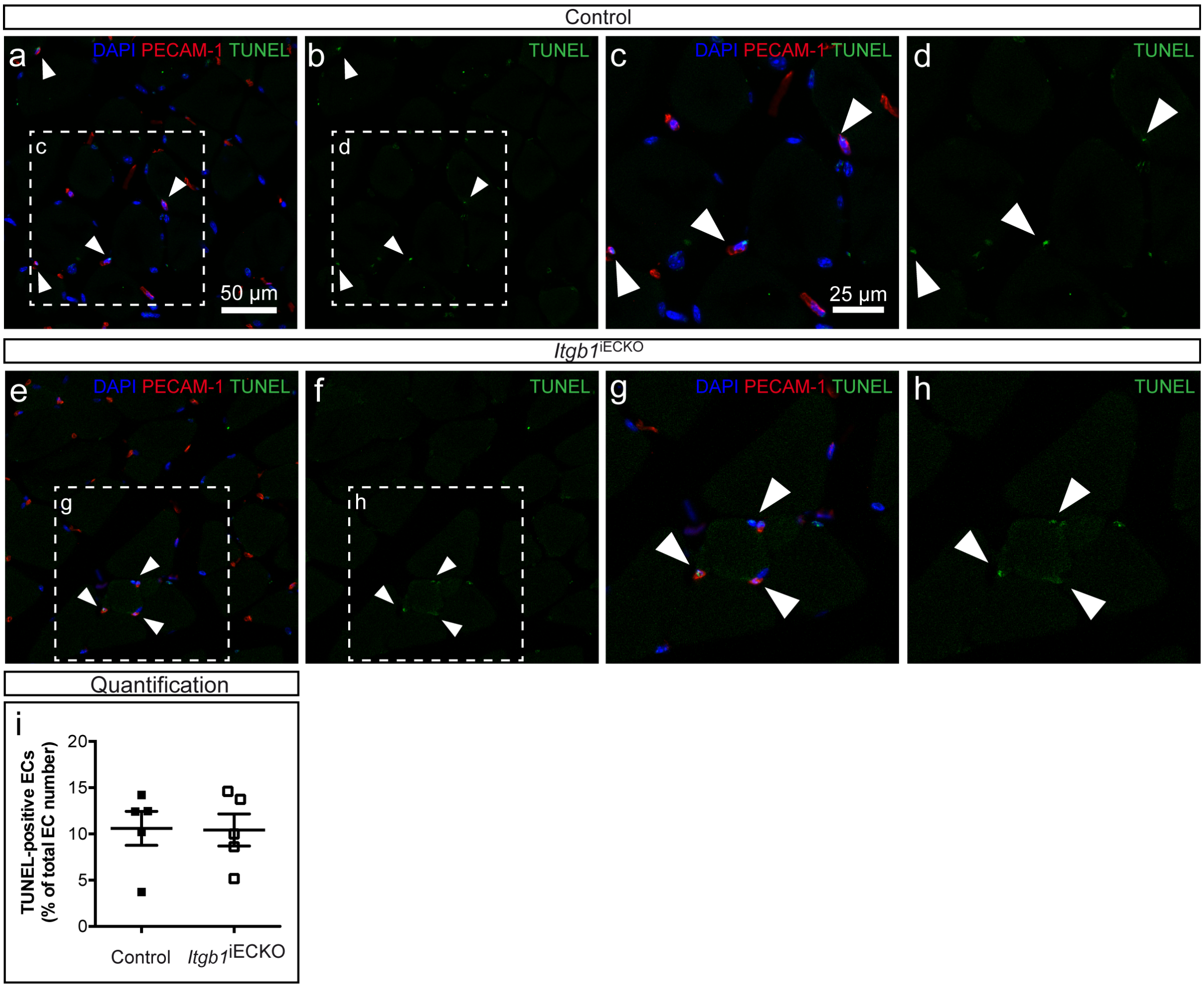

Supplementary Fig. S6

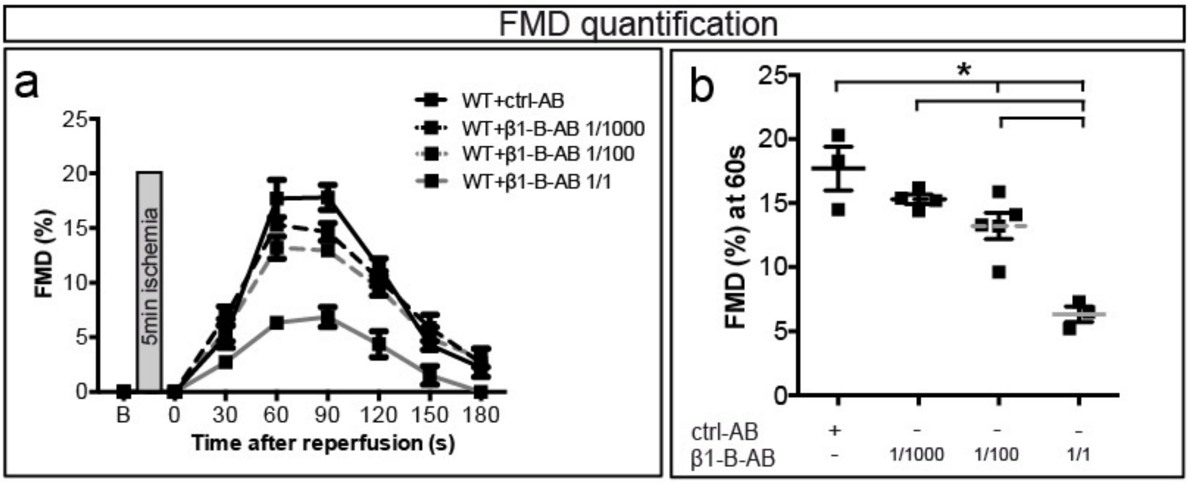

Supplementary Fig. S7

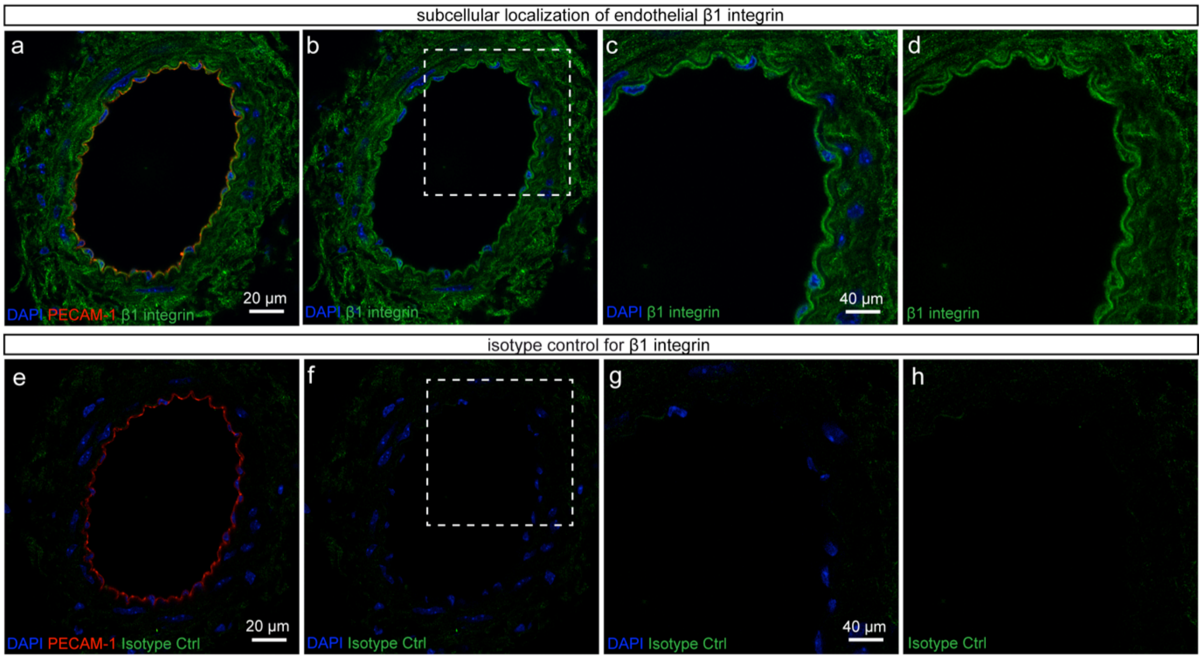

Supplementary Fig. S8

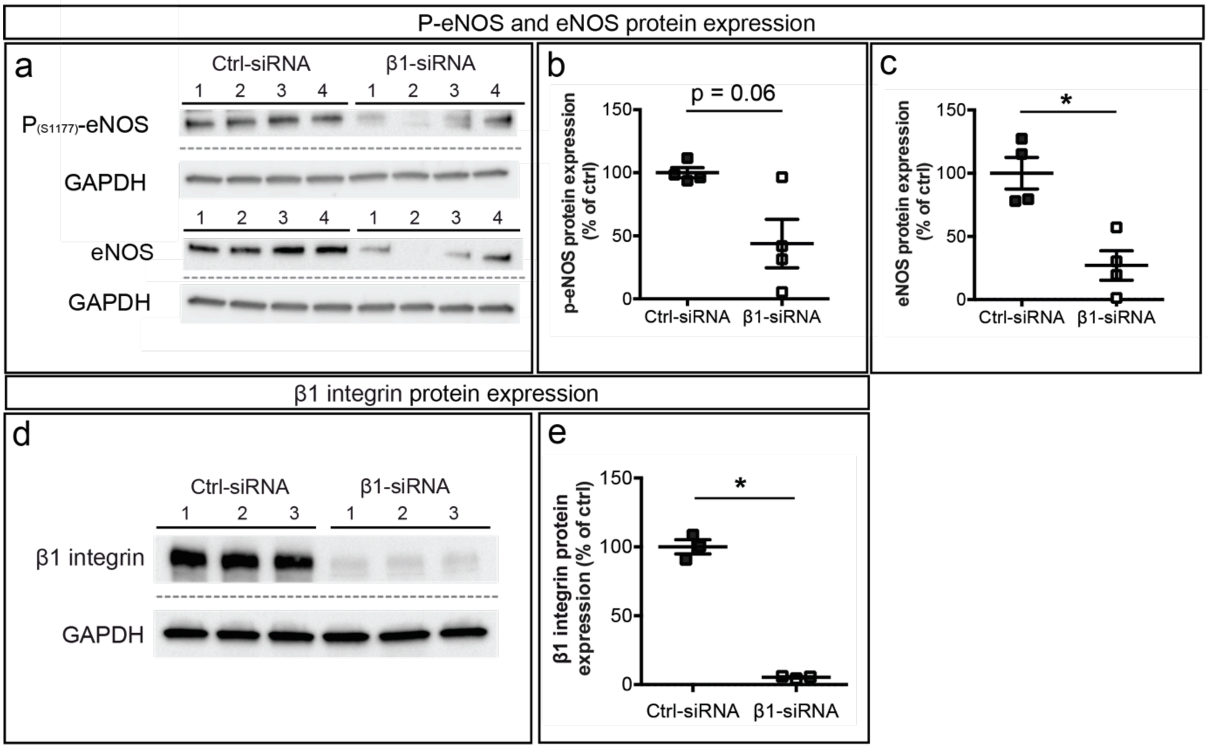

Supplementary Fig. S9

Uncropped Western blot Suppl. Fig. 9a

a **short exposure** **long exposure**

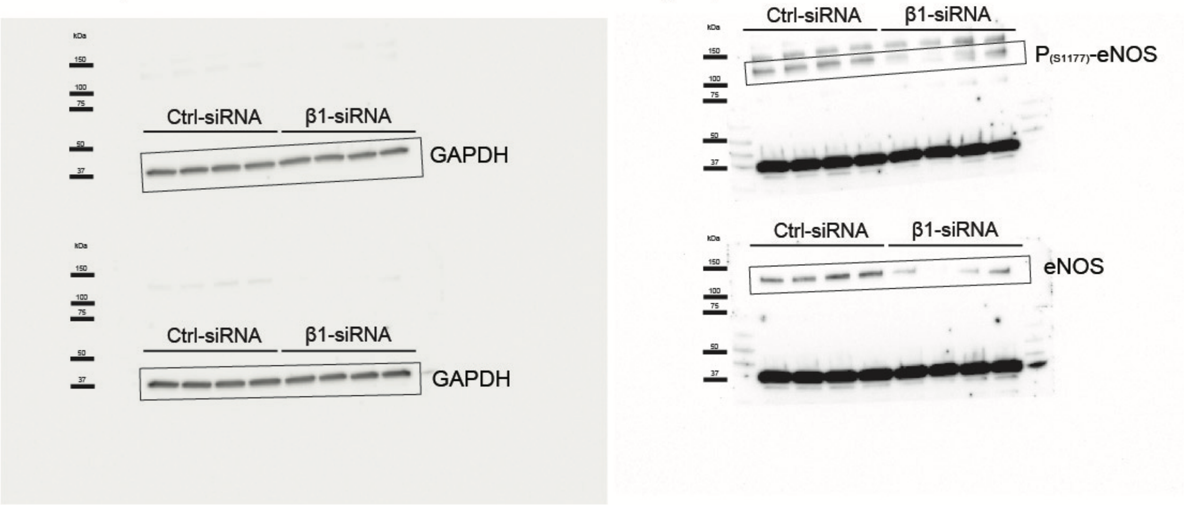

b Uncropped Western blot Suppl. Fig. 9d

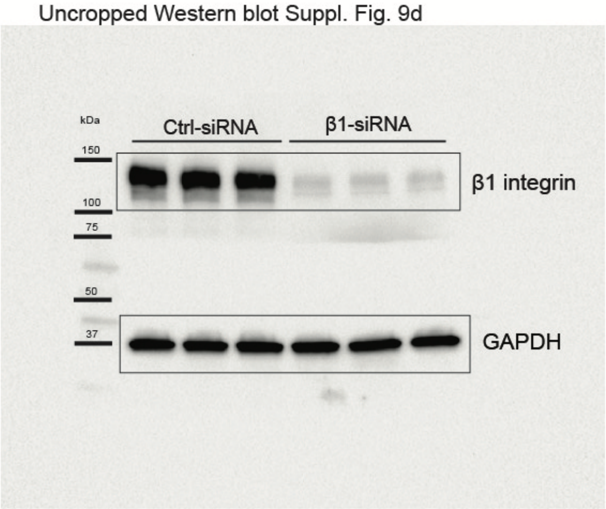

Supplementary Fig. S10
